# Visibility of early gastric cancer in texture and color enhancement imaging

**DOI:** 10.1002/deo2.46

**Published:** 2021-08-24

**Authors:** Seiichiro Abe, Takayuki Yamazaki, Izumi Tanimoto Hisada, Mai Ego Makiguchi, Shigetaka Yoshinaga, Tomoya Sato, Satoru Nonaka, Haruhisa Suzuki, Ichiro Oda, Yutaka Saito

**Affiliations:** ^1^ Endoscopy Division National Cancer Center Hospital Tokyo Japan; ^2^ Department of Advanced Image Processing Technology Olympus Medical Systems Corporation Tokyo Japan; ^3^ Department of Internal Medicine Kawasaki Rinko General Hospital Kanagawa Japan

**Keywords:** early gastric cancer, image enhanced endoscopy, texture and color enhancement imaging, visibility

## Abstract

**Objective:**

There are little data regarding the efficacy of texture and color enhancement imaging (TXI) for early gastric cancer (EGC) diagnosis. This study aimed to compare the color difference and visibility of EGC between white light imaging (WLI) and TXI.

**Methods:**

This study included 20 EGCs of 18 patients undergoing endoscopic submucosal dissection. Still images of EGC in WLI, TXI mode 1 (with color enhancement), and TXI mode 2 (without color enhancement), which were consistent in distance, angle, and air insufflation, were constructed by computer simulation. The center of the lesion, eight equal peripheral points 5 mm outside the lesion, and eight inner points two‐thirds of the distance from peripheral points to the EGC lesion center were annotated. Mean color differences (ΔE) of the area between peripheral and inner points per lesion in WLI, TXI mode 1, and TXI mode 2 were analyzed. In addition, four endoscopists independently scored the visibility of EGC images of TXI mode 1 and 2 compared with WLI.

**Results:**

Clinicopathological characteristics were as follows: 0‐IIa/0‐IIb/0‐IIc/0‐IIa+IIc = 6/1/11/2, reddish/pale = 10/10, differentiated/undifferentiated = 18/2, median tumor size = 13.5 mm. Mean ΔE ± SD = WLI/TXI mode1/TXI mode2 = 10.3 ± 4.7, 15.5 ± 7.8, and 12.7 ± 6.1, respectively. Mean ΔE was significantly higher in TXI mode 1 than in WLI. Visibility (improved/no change/decreased) was 7/13/0 and 4/16/0 in TXI mode 1 and 2, respectively. The visibility was significantly more commonly improved in the macroscopic type 0‐IIc or 0‐IIb than in 0‐IIa or IIa+IIc in TXI mode 1.

**Conclusions:**

TXI could improve the visibility of EGC compared with WLI.

## INTRODUCTION

Gastric cancer is the fifth most common malignant tumor and the third leading cause of cancer‐related death worldwide.^1^ The prognosis of patients with gastric cancer depends on the cancer stage at diagnosis. Although patients with advanced gastric cancer have a poor prognosis, the 5‐year disease‐specific survival rate of patients with early gastric cancer (EGC) is greater than 90%.^2^ Thus, early detection is essential to improve the prognosis of gastric cancer.

Endoscopy has been recently accepted as a primary tool in population‐based gastric cancer screening in countries with high gastric cancer incidence, such as Japan and Korea.^3^, ^4^ However, sufficient training is required to achieve satisfactory EGC detection by screening esophagogastroduodenoscopy (EGD) due to variation in lesion characteristics and discrepancy in the observation of the gastric mucosa between endoscopists. In fact, several studies reported that the false negative rates of gastric cancer during screening EGD ranged from 4.6% to 25.8%.^5^, ^6^, ^7^, ^8^, ^9^, ^10^, ^11^ One of the main reasons for missed gastric cancer is the subtle change of mucosal color and morphology of EGC using conventional white light endoscopy. The utilization of image‐enhanced endoscopy for the detection of EGC is presently under discussion.^12^

Texture and color enhancement imaging (TXI) has been developed to improve the quality of screening EGD.^13^ It is a white light image (WLI)‐based imaged‐enhanced endoscopy that enhances brightness in the dark areas, texture such as subtle surface elevation or depression, and slight color change in the images. There is little clinical data to date regarding the efficacy of TXI enhancement in EGC visibility. This study aimed to compare the color difference and visibility of EGC between WLI and TXI.

## METHODS

### Study subject

This study was a post hoc analysis of a commissioned research at National Cancer Center Hospital, Tokyo, Japan. An expert endoscopist recognized by the Japanese Gastroenterological Endoscopy Society (SA) video‐recorded preoperative endoscopies and stored raw video image data of EGC observation. This study included patients with EGCs for whom raw data were available, and who underwent endoscopic submucosal dissection (ESD) between July 26 and November 30, 2019. Availability of the endoscopy rooms equipped for raw data recording were limited, and the research allowed only one endoscopist to store the raw image data of EGC.

Patients without raw image data were excluded from analysis, either because preoperative EGD was performed by other endoscopists or because it was performed in endoscopy rooms where raw data could not be obtained. All EGCs were clinically diagnosed as gastric adenocarcinoma and met the criteria for ESD according to the Japanese Guidelines as follows: (a) differentiated‐type mucosal cancer without ulceration, or (b) differentiated‐type mucosal cancer ≤ 3 cm in diameter with ulceration, or (c) undifferentiated‐type mucosal cancer ≤ 2 cm without ulceration.^14^ Gastric atrophy was graded according to the Kimura‐Takemoto Classification.^15^ The study was conducted in accordance with the ethical principles included in the Declaration of Helsinki. We obtained approval from the institutional review board, and written informed consent was obtained from all patients. This study was registered with the University hospital Medical Information Network (UMIN000043838).

### TXI

**FIGURE 1:**
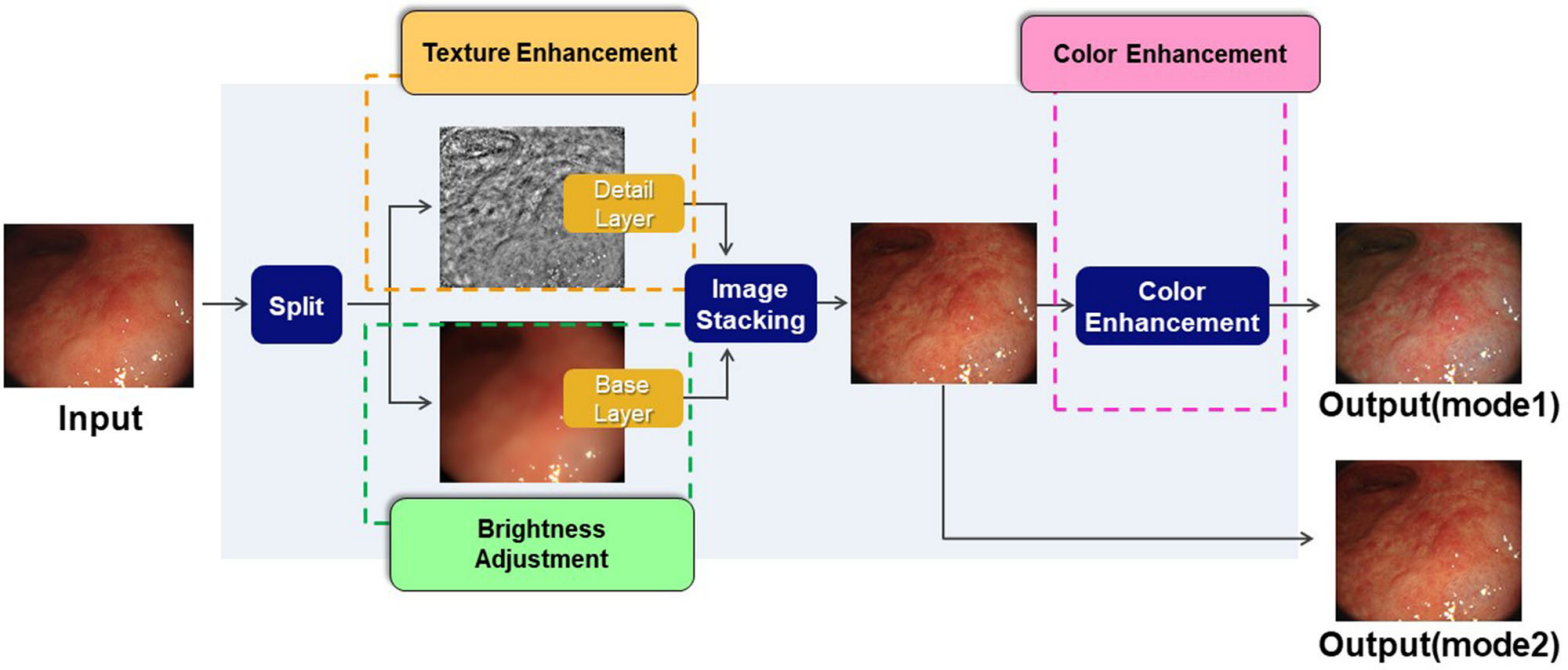
Concept of texture and color enhancement imaging (TXI). A white light image is decomposed into the detail layer and the base layer. Texture of an image such as subtle surface elevation or depression is strengthened by enhancing the detail layer. The partial brightness control in the image can be achieved by processing techniques to adjust the brightness of the base layer. The enhanced detail layer and the corrected base layer are recombined to produce one of the TXI outputs. In addition, the color tone of that output is highly enhanced, which is designed to exaggerate the color difference, especially between red and white colors. There are two modes in TXI: mode 1 is one which outputs with color enhancement. The other is mode 2 which outputs without color enhancement, similar to the white‐light color tones

TXI is a novel image‐enhanced endoscopy modality developed by Olympus Corporation (Tokyo, Japan).^13^ It enhances three aspects of white light endoscopy: (a) brightness in the dark areas, (b) texture such as subtle surface elevation or depression, and (c) color such as slight color changes in the images. It utilizes an image processing technology which is based on the Retinex theory.^16^ Applying its concept, an image is decomposed into two layers according to the working principles of the human visual system. One is a detail layer corresponding to the local contrast of brightness and color in the scene. The contrast enhancement of slight morphology or color change is achieved in the detail layer. The other is a base layer which represents the illumination component in the scene. It is difficult to adjust the brightness selectively in dark regions of an image by light source control, because light can only be applied across the entire image resulting in halation of the brighter areas. On the other hand, since the base layer corresponds to the illumination component, partial brightness control in the image can be achieved through processing techniques. The enhanced detail layer and the corrected base layer are recombined to produce one of the TXI outputs. In addition, the color tone of that output is highly enhanced, exaggerating the color difference in the images particularly between red and white hues.^17^ There are two settings for TXI: mode 1 with color enhancement, and mode 2 without color enhancement. Mode 2 produces images closer to WLI color tone (Figure 1).

### Image data collection

The expert endoscopist performed the preoperative endoscopy of EGCs with a high definition gastroscope, GIF‐H290Z and LUCERA ELITE system (Olympus Corporation), for patients sedated with intravenous propofol. Magnifying endoscopy with narrow‐band imaging was also applied for the detailed assessment of the EGC margin.^18^ All preoperative endoscopies were video‐recorded, and preprocessed raw image data (i.e., image sensor output) were obtained. The expert endoscopist selected one representative WLI from the recorded video which included an entire EGC lesion. The raw data of the representative image were then extracted, and the still images of EGC in WLI, TXI mode 1, and TXI mode 2 consistent in distance, angle, and air insufflation were constructed by computer simulation. All the simulations were conducted on a PC running Windows 10 professional 64‐bit system utilizing Microsoft Visual Studio C++ 2012. Gastric ESD was performed according to the guidelines standard.^14^ The resected ESD specimens were fixed in 10% formalin before histopathological assessment. Histological mapping was performed after the specimens were serially sectioned at 2 mm intervals. Certified pathologists at our institution assessed the histological types, macroscopic appearance, tumor size, depth of invasion, presence of ulceration, lymphovascular invasion, and margin of all lesions according to the Japanese classification of gastric carcinoma.^19^

### Annotation of lateral margin, peripheral, and inner points on EGC images

**FIGURE 2:**
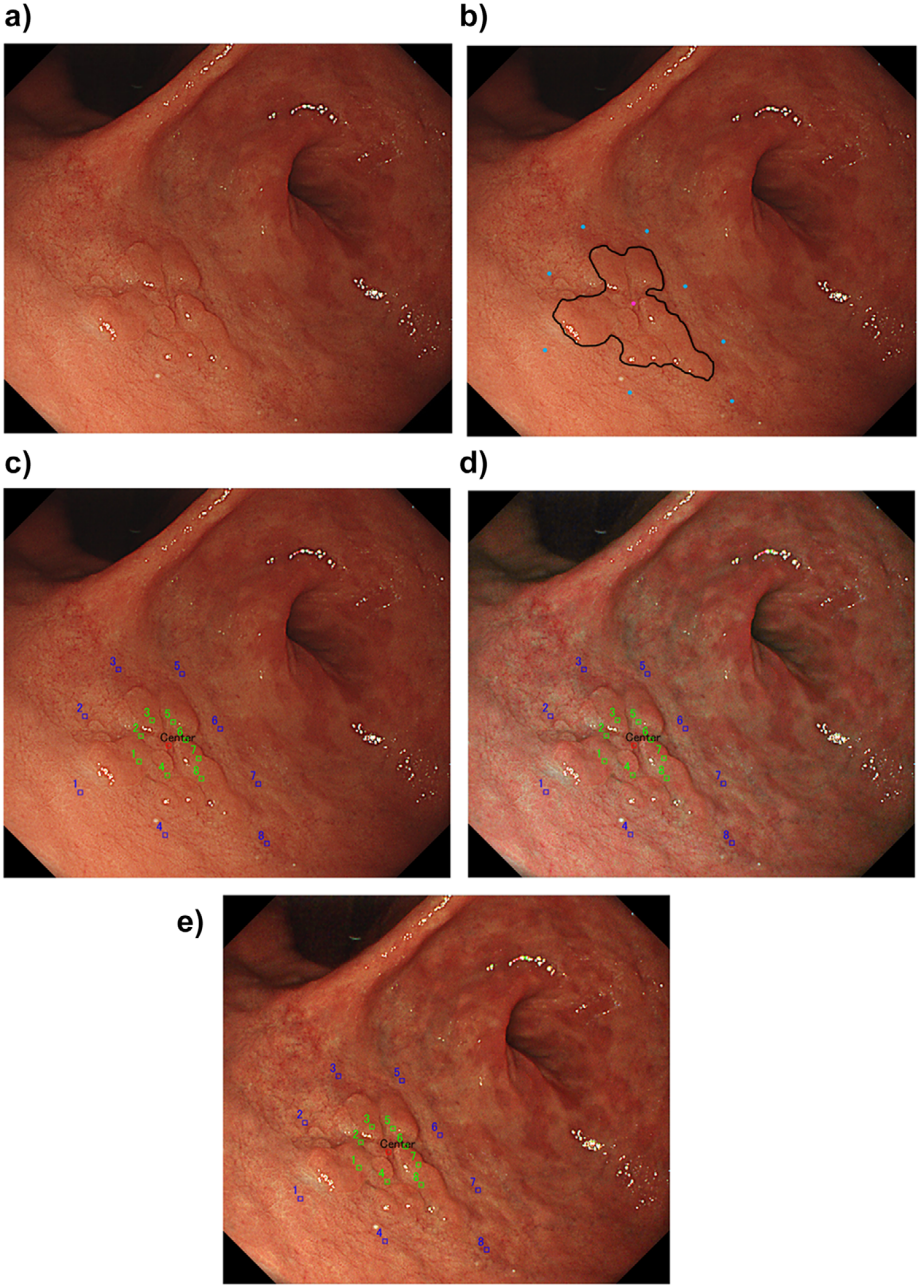
Annotation of lateral margin, peripheral, and inner points on EGC images for color difference analysis. (a) A reddish elevated lesion is seen in the anterior wall of the antrum. (b) An expert endoscopist defined the region of interest and delineated the margin of EGC on the WLI image. The endoscopist manually annotated the center of the lesion, eight equal peripheral non‐cancerous points 5 mm outside the lesion (proximal, distal, anterior, posterior, and four midpoints between the two points). (c) Eight innerpoints (green spots) were annotated two‐thirds of the distance from peripheral points to the EGC lesion center. Blue spots indicate the peripheral points. (d) The peripheral and inner points were similarly annotated on the image of TXI mode 1 with the same angle, distance, and air insufflation. (e) The peripheral and inner points were similarly annotated on the image of TXI mode 2 with the same angle, distance, and air insufflation

The expert endoscopist defined the region of interest and delineated the margin of EGC on the WLI images based on magnifying endoscopy with NBI and matched histology mapping with markings at ESD. The endoscopist then manually annotated the center of the lesions, eight equal peripheral non‐cancerous points at 10 × 10 pixels 5 mm outside the lesions (proximal, distal, anterior, posterior, and four midpoints between the two points), and eight inner cancerous points at 10 × 10 pixels two‐thirds of the distance from peripheral points to the EGC lesion center (Figures 2a‐2c). The eight equal peripheral points and eight inner points were similarly annotated on TXI mode 1 and mode 2 (Figures 2d and 2e).

### The color difference between **peripheral and inner points on the EGC image**

The color in the endoscopic images was evaluated using the International Commission on Illumination L*a*b* (CIELAB) color space system.^17^ It is a three‐dimensional space for presenting a color with axes of L* (from black to white; white is highest), a* (from green to red; red is highest), and b* (from blue to yellow; yellow is highest). The distance between two points in the CIELAB color space is proportional to the difference in color perception. Here, we assessed the perceived color difference of the area between peripheral points and inner points with the formula: ΔE = [(ΔL*)2 + (Δa*)2 + (Δb*)2]1/2. The color difference analysis did not include the pairs between the peripheral and inner points if the inner points were annotated on the non‐neoplastic area. We analyzed the color difference between peripheral points and inner points using the pairs where the data were available in all EGCs. Moreover, we performed a subanalysis of the mean color difference for EGCs in which the data were available in all eight pairs. Mean color differences of the area between peripheral and inner points per lesion (mean ΔE) were analyzed in WLI, TXI mode 1, and TXI mode 2. ΔE was measured using MATLAB 2019b (The MathWorks, Inc., Massachusetts, USA).

### Visibility assessment of EGC

Two expert endoscopists and two non‐expert endoscopists evaluated the visibility of EGC. The former expert endoscopist who annotated the peripheral and inner points did not participate in this analysis. The four endoscopists independently reviewed the image data set consisting of 40 paired endoscopic images (20 WLI and TXI mode 1, and 20 WLI and TXI mode 2), which were randomly shown on a laptop monitor. They scored the visibility of EGC images of TXI mode 1 and 2 compared with WLI. Visibility of EGC was scored according to the following scale: +2 (improved visibility), +1 (somewhat improved visibility), 0 (visibility equivalent to that of WLI), −1 (somewhat decreased visibility), and −2 (decreased visibility). The improved visibility was defined as a total score of 5 or more, no change between −4 and 4 and decreased visibility as −5 or less, according to a previous study by Imagawa et al.^20^

### Statistical analysis

Quantitative variables were shown as a mean with standard deviation and were analyzed using the Bonferroni test. A *p* value < 0.05 was considered statistically significant. All statistical analyses were performed with EZR (Saitama Medical Center, Jichi Medical University, Saitama, Japan), which is a graphical user interface for R (The R Foundation for Statistical Computing, Vienna, Austria).^21^

## RESULTS

**FIGURE 3:**
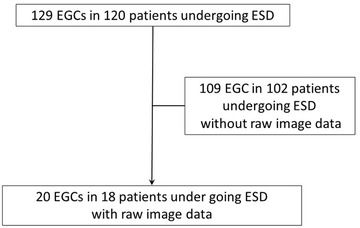
The patient flow of this study. Among 129 EGCs in 120 patients undergoing ESD, a total of 20 EGCs in 18 patients were video‐recorded and evaluated in this study. One hundred two patients without raw image data were excluded from analysis

Among 129 EGCs in 120 patients undergoing ESD, a total of 20 EGCs in 18 patients (13 male and five female with median age [range] of 72 [39–83] years) were video‐recorded and evaluated in this study. One hundred two patients without raw image data were excluded from analysis (Figure 3). The predominant macroscopic appearance was Type 0‐IIc followed by Type 0‐IIa, and the median tumor size (range) was 13.5 (2–32) mm. Ten lesions were reddish, and 10 were pale in color compared with background gastric mucosa. The majority of the lesions were characterized as differentiated‐type mucosal EGC post *H. pylori* eradication. All EGC were resected en‐bloc via ESD with adequate lateral and horizontal margin (Table 1).

**TABLE 1:** Patient and lesion characteristics

Gender, male/female, *n*	13/5
Median age (range), year	72 (39–82)
Gastric atrophy^*^ (C‐1∼2/C‐3,O‐1/O‐2∼3)	4/4/10
*H. pylori* infection	
Positive/negative/eradicated, *n*	2/4/12
Lesion location, upper/middle/lower, *n*	10/7/3
Median tumor size (range), mm	13.5 (2–32)
Macroscopic type	
0‐IIa/0‐IIb/0‐IIc/0‐IIa+IIc, *n*	6/1/11/2
Color (reddish/pale), *n*	10/10
Histological type	
Differentiated/Undifferentiated, *n*	18/2
Depth of invasion, M/SM1, *n*	19/1

Abbreviations: M, mucosa; SM1, superficial submucosa < 500μm.

^*^ Graded by Kimura Takemoto Classification.

The color difference was evaluated in all eight paired points in 13 EGCs. It could be analyzed in seven paired points in two EGCs, six paired points in one EGC, five paired points in one EGC, four paired points in two EGCs, and one paired points in one EGC, as the inner points where annotated on the non‐neoplastic area were not evaluated. The color difference could be evaluated between the peripheral and inner points only at the distal side where the data were available in all 20 EGCs. The mean Δ ± SD of the distal side in WLI, TXI mode 1, and TXI mode 2 was 10.3 ± 4.7, 15.5 ± 7.8, and 12.7 ± 6.1, respectively. The mean ΔE was significantly higher in TXI mode 1 than in WLI (*p* = 0.04). There was no significant difference in the mean ΔE between TXI mode 1 and 2 (*p* = 0.54) and between WLI and TXI mode 2 (*p* = 0.61) (Table 2). Moreover, we also performed a subanalysis of the color difference in the 13 EGCs in which the data were available in all eight pairs. The mean Δ ± SD in WLI, TXI mode 1, and TXI mode 2 was 10.1 ± 3.0, 13.5 ± 3.4, and 11.3 ± 2.6, respectively. The mean ΔE was significantly higher in TXI mode 1 than in WLI (*p* = 0.04). There was no significant difference in the mean ΔE between TXI mode 1 and 2 (*p* = 0.24) and between WLI and TXI mode 2 (*p* = 0.86).

**TABLE 2:** Color difference between peripheral and inner points (*n* = 20)

	WLI	TXI mode 1	TXI mode2	*p* values (WLI vs. TXI mode1/ TXI mode 1 vs. 2/ WLI vs. TXI mode 2)		
mean Δ ± SD	10.3 ± 4.7	15.5 ± 7.8	12.7 ± 6.1	0.04	0.54	0.61

Abbreviations: SD, standard deviation; TXI, texture and color enhancement imaging; WLI, white light imaging.

**FIGURE 4:**
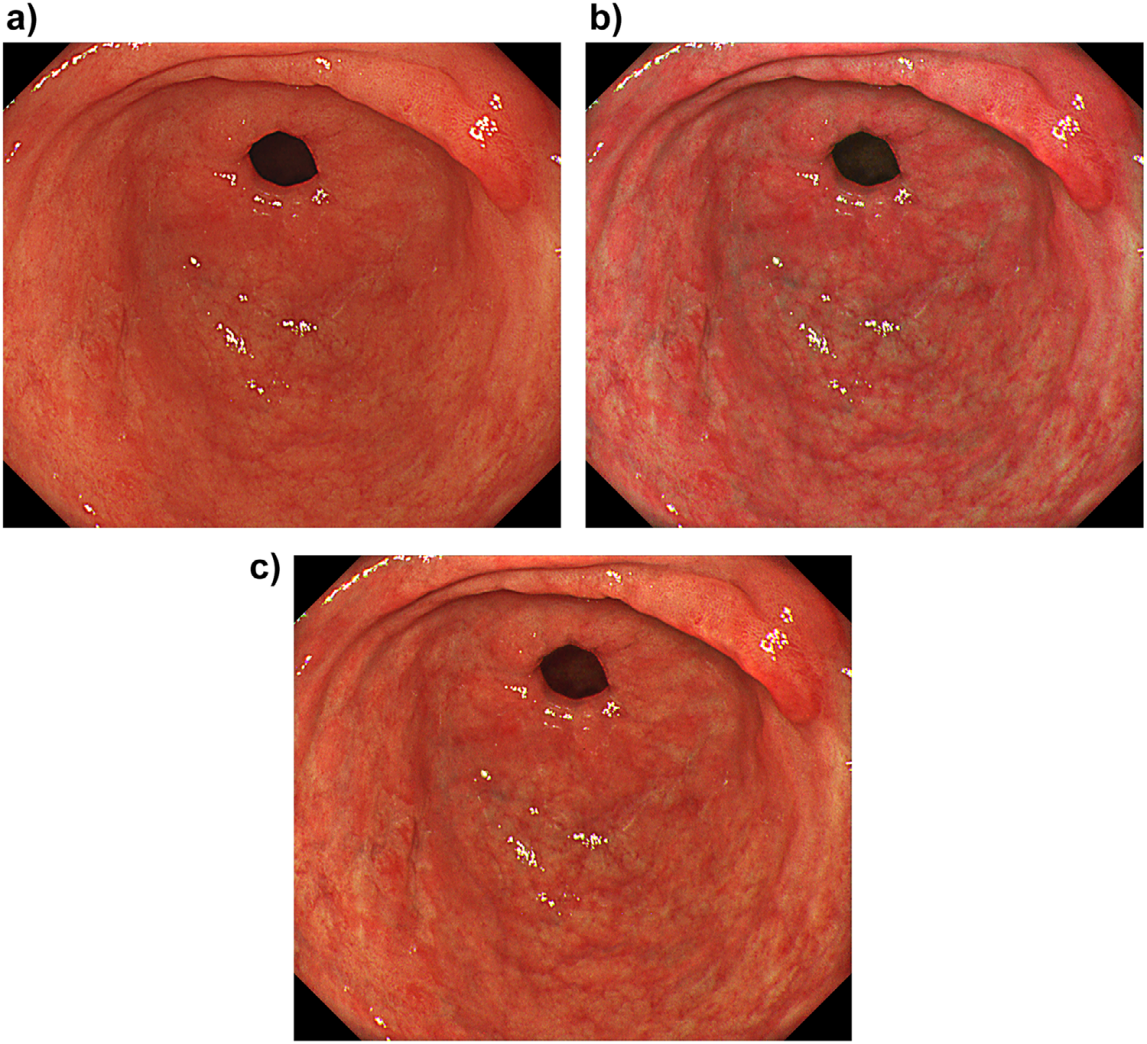
A representative case of EGC with improved visibility. (a) WLI showed a slightly pale lesion in the anterior wall of the antrum. The mean ΔE was 4.34. (b) TXI mode 1 demonstrated enhancement of the depression and pale color with a mean ΔE of 7.18. The visibility was scored as improved, with a total visibility score of 6. (c) TXI mode 2 demonstrated enhancement of the depression with a mean ΔE of 6.04. The visibility was scored as improved, with a total visibility score of 7

The visibility scores rated by the four endoscopists are shown in Table 3. The mean total visibility scores were 3.4 ± 2.0 and 3.0 ± 1.9 in TXI mode 1 and 2, respectively. Visibility was improved in 35% (7/20) and 20% (4/20), and unchanged in 65% (13/20) and 80% (16/20) in TXI mode 1 and 2 compared with WLI, respectively. None of the lesions were scored as decreased visibility. The mean visibility scores between non‐expert and expert endscopists were 1.6 versus 1.9 and 1.5 versus 1.5 in TXI mode 1 and TXI mode 2, respectively. The visibility was significantly more commonly improved in the macroscopic type 0‐IIc or 0‐IIb than in 0‐IIa or IIa+IIc in TXI mode 1 (*p* = 0.04) (Table 4, Figure 4). There was no lesion characteristic associated with improved visibility in TXI mode 2 (Table 5).

**TABLE 3:** Visibility score (*n* = 20)

	Visibility score (mean ± SD)	Visibility compared with WLI, % (n)		
		Improved	Equivalent	Decreased
TXI mode 1	3.4 ± 2.0	35 (7)	65 (13)	0
TXI mode 2	3.0 ± 1.9	20 (4)	80 (16)	0

**TABLE 4:** Lesion characteristics with and without improved visibility in TXI mode1

		Improved	No change	
		*n* = 7	*n* = 13	*p* value
Location	U/ML, % (*n*)	0/100	38.5/61.5	0.11
		(0/7)	(5/8)	
Color	Reddish/pale, % (*n*)	28.6/71.4	61.5/38.5	0.35
		(2/5)	(8/5)	
Macroscopic type	0‐IIc,0‐IIb/0‐IIa,IIa+IIc, % (*n*)	100/0	38.5/61.5	0.01
		(7/0)	(5/8)	
Histology	D‐type/UD‐type, % (*n*)	85.7/14.3	92.3/7.7	1.0
		(6/1)	(12/1)	
Size	≤10mm/>11mm, % (*n*)	57.1/42.9	46.2/53.8	1.0
		(4/3)	(6/7)	
Depth of invasion	M/SM, % (*n*)	100/0	92.3/7.7	1.0
		(7/0)	(12/1)	
*H.pylori* infection	Positive/Eradicated/Negative, % (*n*)	0/71.4/28.6	15.4/69.2/15.4	0.52^*^
		(0/5/2)	(2/9/2)	

^*^ Positive versus eradicated, negative.

Abbreviations: D‐type, differentiated type; M/SM, mucosa/submucosa; UD‐type, undifferentiated type; U/M/L, upper third/middle third/lower third.

**TABLE 5:** Lesion characteristics with and without improved visibility in TXI mode 2

		Improved	No change	
		*n* = 4	*n* = 16	*p* value
Location	U/ML, % (*n*)	0/100	18.7/81.3	1.0
		(0/4)	(3/13)	
Color	Reddish/pale, % (*n*)	25/75	56.2/43.8	0.58
		(1/3)	(9/7)	
Macroscopic type	0‐IIc,0‐IIb/0‐IIa,IIa+IIc, % (*n*)	100/0	50/50	0.12
		(4/0)	(8/8)	
Histology	D‐type/UD‐type, % (*n*)	100/0	87.5/12.5	1.0
		(4/0)	(14/2)	
Size	≤10mm/>11mm, % (*n*)	50/50	50/50	1.0
		(2/2)	(8/8)	
Depth of invasion	M/SM, % (*n*)	100/0	93.8/6.2	1.0
		(4/0)	(15/1)	
*H. pylori* infection	Positive/Eradicated/Negative, % (*n*)	0/75/25	12.5/68.8/18.7	1.0^*^
		(0/3/1)	(2/11/3)	

^*^ Positive versus eradicated, negative.

Abbreviations: D‐type, differentiated type; M/SM, mucosa/submucosa; UD‐type: undifferentiated type; U/M/L, upper third/middle third/lower third.

## DISCUSSION

This study investigated the color difference (mean ΔE) between non‐neoplastic and neoplastic areas of the stomach comparing WLI, TXI mode 1, and TXI mode 2. It revealed that the mean ΔE was significantly higher in TXI mode 1 than in WLI. The results were consistent with the TXI algorithm developed by Sato,^13^ and we validated the preclinical data from the initial proof of concept study. The EGC visibility scored by four endoscopists improved in 35% of TXI mode 1 and 20% of TXI mode 2, respectively. It is important to pay attention to subtle changes of mucosal color and morphology in detecting EGC during screening endoscopy. Clues to a suspicious lesion include mucosal discoloration (erythema or pallor) and morphologic changes of the mucosal surface (protruding, elevated, or depressed).^22^ Thus, the concept of TXI to improve the visibility of EGC by enhancing subtle color and morphological differences on gastric mucosal compared with conventional WLI is plausible. This study indicated that TXI could improve endoscopic visibility of EGC, potentially increasing EGC detection during screening EGD. Notably, the visibility was significantly more commonly improved in the macroscopic type 0‐IIc or 0‐IIb than 0‐IIa or 0‐IIa+IIc. This is worthwhile noting when considering the application of TXI in daily clinical practice because 0‐IIc is the most major macroscopic type of EGC.

To the best of our knowledge, there was only one previous study that reported the visibility of EGC in TXI. Ishikawa et al recently investigated the color difference between non‐neoplastic and neoplastic areas of 12 gastric neoplasms in WLI and TXI. The color differences ± SD in WLI, TXI mode 1, and TXI mode 2 were 8.000 ± 4.263, 18.728 ± 16.046, and 10.246 ± 8. 379, respectively. The color difference was significantly higher in TXI mode 1 than WLI and TXI mode 2. In addition, the visibility of gastric neoplastic lesion (gastric adenocarcinoma and adenoma) evaluated by six endoscopists was better in TXI mode 1 compared with WLI.^23^ The reason for inconsistent results could be explained by the image data. They manually annotated a couple of inner and peripheral points for each image. It would have been very challenging during endoscopy to capture still images of EGC in WLI, TXI mode 1, and TXI mode 2 at exactly the same distance, angle, and air insufflation. These factors could influence the results of their study. In our study, we constructed still images of TXI mode 1 and TXI mode 2 from WLI images consistent in distance, angle, and air insufflation, using computer simulation. The color difference was evaluated in only one pair where the data were available in all 20 EGCs, as done by Ishikawa et al. However, the data of our study were reinforced by the subanalysis using 13 EGCs in which the mean color difference could be analyzed in all eight pairs. Because the annotation can greatly influence the data interpretation of color difference between non‐neoplastic and neoplastic areas, we believe our study demonstrated more objective data analysis of color difference than the former study.

Other image‐enhanced endoscopies could improve visibility and EGC detection rate during screening EGD.^24^ Among them, blue light imaging made from the combination of strong laser light with a 410 nm wavelength and weak laser light with a 450 nm wavelength, was reported to have a higher real‐time EGC detection rate than WLI.^25^ Linked color imaging (LCI) is another color enhancement technology that provides enhanced color differences in mucosal color, allowing better lesion recognition with sufficient brightness compared with BLI. It is a similar image enhanced technology to TXI, and a randomized controlled trial showed LCI is more effective than WLI for detecting neoplastic lesions in the pharynx, esophagus, and stomach (4.8% vs. 8.0%, *p* = 0.011).^26^ However, there was no statistically significant difference in the gastric neoplastic lesion detection rates (3.3% vs. 5.5%), perhaps owing to insufficient sample size. The primary endpoint included pharyngeal and esophageal neoplastic lesions as well as gastric neoplastic lesion. Another randomized controlled trial by Yoshida et al compared the EGC detection rate between WLI and second‐generation NBI and concluded that second‐generation NBI did not increase EGC detection compared with WLI.^27^ The Japanese Guidelines for Endoscopic Diagnosis of Early Gastric Cancer stated that the utility of image‐enhanced endoscopy for detecting EGC remains unclear.^12^ The results of this study were not relevant to gastric cancer detection. The skill and experience of endoscopist are also associated with the detection. The potential of TXI, consisting of the combination of texture and color enhancement, should be further evaluated. Large clinical trials are warranted to investigate the performance of TXI during real‐time EGC screening.

There were some limitations in this study. Firstly, the sample size was small, and selection bias could have influenced the results. *H. pylor*i infection status may affect the visibility of TXI. However, we could not sufficiently evaluate it. Secondly, this study utilized image‐based analysis, and TXI was not performed in real clinical setting but constructed with computer simulation. Conversely, a more objective image analysis was achieved, as TXI and WLI images uniform in distance, angle, and air insufflation were used, rather than images taken from real clinical setting where consistent images across the modality would not be possible. Thirdly, the endoscopists were not blinded whether an image was WLI or TXI. Finally, this study did not analyze the visibility of non‐cancerous lesions. Further investigation is required in clinical practice.

In conclusion, TXI could improve the visibility of EGC compared with WLI.

## CONFLICT OF INTEREST

The author Seiichiro Abe was supported by a research grant from Olympus Medical Systems (C2020‐036). The authors Seiichiro Abe and Yutaka Saito received honoraria for lectures from Olympus Medical Systems. The author Seiichiro Abe is an associate editor of DEN Open.

## FUNDING INFORMATION

The author Seiichiro Abe was supported by a research grant from Olympus Medical Systems (C2020‐036).
